# The Effects of Cognitive Therapy Versus ‘Treatment as Usual’ in Patients with Major Depressive Disorder

**DOI:** 10.1371/journal.pone.0022890

**Published:** 2011-08-04

**Authors:** Janus Christian Jakobsen, Jane Lindschou Hansen, Ole Jakob Storebø, Erik Simonsen, Christian Gluud

**Affiliations:** 1 Psychiatric Research Unit, Copenhagen University Hospital, Region Zealand, Roskilde, Denmark; 2 Copenhagen Trial Unit, Department 3344 Rigshospitalet, Centre for Clinical Intervention Research, Copenhagen University Hospital, Copenhagen, Denmark; Charité-Universitätsmedizin Berlin, Germany

## Abstract

**Background:**

Major depressive disorder afflicts an estimated 17% of individuals during their lifetimes at tremendous suffering and costs. Cognitive therapy may be an effective treatment option for major depressive disorder, but the effects have only had limited assessment in systematic reviews.

**Methods/Principal Findings:**

Cochrane systematic review methodology, with meta-analyses and trial sequential analyses of randomized trials, are comparing the effects of cognitive therapy versus ‘treatment as usual’ for major depressive disorder. To be included the participants had to be older than 17 years with a primary diagnosis of major depressive disorder. Altogether, we included eight trials randomizing a total of 719 participants. All eight trials had high risk of bias. Four trials reported data on the 17-item Hamilton Rating Scale for Depression and four trials reported data on the Beck Depression Inventory. Meta-analysis on the data from the Hamilton Rating Scale for Depression showed that cognitive therapy compared with ‘treatment as usual’ significantly reduced depressive symptoms (mean difference −2.15 (95% confidence interval −3.70 to −0.60; P<0.007, no heterogeneity)). However, meta-analysis with both fixed-effect and random-effects model on the data from the Beck Depression Inventory (mean difference with both models −1.57 (95% CL −4.30 to 1.16; P = 0.26, I^2^ = 0) could not confirm the Hamilton Rating Scale for Depression results. Furthermore, trial sequential analysis on both the data from Hamilton Rating Scale for Depression and Becks Depression Inventory showed that insufficient data have been obtained.

**Discussion:**

Cognitive therapy might not be an effective treatment for major depressive disorder compared with ‘treatment as usual’. The possible treatment effect measured on the Hamilton Rating Scale for Depression is relatively small. More randomized trials with low risk of bias, increased sample sizes, and broader more clinically relevant outcomes are needed.

## Introduction

According to the WHO, major depressive disorder is the second largest healthcare problem worldwide in terms of disability caused by illness [1]. It afflicts an estimated 17% of individuals during their lifetimes at tremendous cost to the individual and society [2], [3], and roughly a third of all depressive disorders take a chronic course [4], [5]. Compared to other medical disorders, major depressive disorder causes the most significant deterioration in individual life quality [6]. Approximately 15% of depressive patients will commit suicide over a 10 to 20 year period [7].

Antidepressant medication remains the mainstay in the treatment of depression [8]. However, meta-analyses have shown that newer antidepressants presumably only obtain beneficial effect in severely depressed patients, and this effect seems to be clinically small [9], [10]. Antidepressants might, however, decrease the risk of relapse [11]. The therapeutic benefits of antidepressants seem to be limited and this raises the question if there are other effective treatments for this serious illness?

Aaron T. Beck originally developed cognitive therapy for depression [12]. Beck believed that critical life events could accentuate hidden negative beliefs, which could generate negative automatic thoughts. These negative thoughts could lead to symptoms of depression, which then could reinforce more negative automatic thoughts. The main goal of the ‘cognitive model of depression’ is to correct these negative beliefs and thoughts in order to treat the depressive symptoms [12]. A Cochrane review shows that cognitive therapy has a preventive effect against recurrent depression, and that this effect clearly surpasses the preventive effects of antidepressant medication [13]. Furthermore, cognitive therapy appears to be an effective treatment for major depressive disorder [14], but we were unable to find any meta-analysis with Cochrane methodology [15] examining the effect of cognitive therapy versus ‘treatment as usual’ for major depressive disorder.

## Methods

We conducted our systematic review of randomized clinical trials involving meta-analysis [15] and trial sequential analysis [16], [17] to answer the question: what are the beneficial and harmful effects of cognitive therapy versus ‘treatment as usual’ in the treatment of major depressive disorder? We used assessment of bias risk to reduce systematic errors [15], and trial sequential analysis to reduce the risk of random errors [16], [17].

For details regarding the methodology please consult our protocol published on our website (www.ctu.dk) in February 2010 before we began systematic literature searches in all relevant databases, data-extraction, and analysis [18].

In short, we included all randomized clinical trials comparing the effect of cognitive therapy alone or in combination with any co-intervention versus ‘treatment as usual’ alone or in combination with a similar co-intervention. These co-interventions had to be administered equally in both intervention groups. The trials were included irrespective of language, publication status, publication year, and publication type - based on searches in The Cochrane Library's CENTRAL, MEDLINE via PubMed, EMBASE, Psychlit, PsycInfo, and Science Citation Index Expanded. The timeframe for the search was all trials published before February 2010.

To be included, participants had to be older than 17 years, with a primary diagnosis of major depressive disorder. Trials were only included if the diagnosis of depression was based on one of the standardized criteria, such as ICD 10 [19], DSM III [20], DSM III-R [21], or DSM IV [22]. Comorbidity with other psychiatric diagnoses was not an exclusion criterion. The following types of trials were excluded:

- Trials focusing on depressed participants with comorbid serious somatic illness, e.g., myocardial infarction, multiple sclerosis, cerebral stroke, cancer, etc.
- Trials focusing on ‘late life’ depression or depression in the elderly, most often participants over 65 years.
- Trials focusing on pregnancy related depression, e.g., postpartum depression, postnatal depression, etc.
- Drug or alcohol dependence related depression.

These exclusions were conducted because we expect participants in such trials to respond differently to standardized psychotherapy than other depressed patients, and these types of depressed patients are traditionally examined in separate trials [23]–[26].

### Interventions

#### Cognitive therapy

Cognitive therapy and cognitive-behavioral therapy are collective terms for a range of different forms of interventions and it is difficult to find a simple definition, which adequately describes this psychotherapeutic method. However, we selected the following criteria from Beck et al., 1979 as being necessary for the intervention to be classified as ‘cognitive therapy’ [12]:

1. That the intervention seeks to link thoughts, feeling and behavior, and relates these to the depressive symptoms.
2. That the intervention seeks to record and correct irrational thoughts or behavioral patterns, and relates this to the depressive symptoms.
3. That the intervention seeks to teach the patient alternative methods of thinking or behaving, and to be able to relate this to the depressive symptoms.
4. That the intervention is undertaken face-to-face either individually or in a group.

We accepted any co-intervention to cognitive therapy as long as this co-intervention was similar and administered equally to the experimental group (cognitive therapy) and the control group (‘treatment as usual’).

Furthermore, the trials had to present a treatment manual and had to document adherence to the treatment manual in order for the intervention to be classified as ‘adequately defined’. All other trials using the term ‘cognitive therapy’ or ‘cognitive-behavioral therapy’ were included, but the intervention was classified as ‘not adequately defined’.

#### ‘Treatment as usual’

For ‘treatment as usual’ control interventions we accepted any non-specific treatments described as: ‘treatment as usual’, ‘standard care’, or ‘clinical management’, etc. To be included the ‘treatment as usual’ intervention had to include some kind of non-specific supportive treatment.

We accepted any co-intervention to ‘treatment as usual’ as long as this co-intervention was similar and administered equally to the experimental group (cognitive therapy) and the control group (‘treatment as usual’).

### Trial selection

Three of the review authors (JJ, JLH, and OJS) independently selected relevant trials. If a trial was selected by three or two of the three, it was included. If a trial was identified only by one of the three, it was discussed whether the trial should be included. Excluded trials were entered on a list, stating the reason for exclusion.

### Data extraction

Data were extracted for trial design, bias risk, and outcomes independently by two authors (JJ and JLH). Disagreements were resolved by discussion or through arbitration (CG). We used the instructions in The Cochrane Handbook for Systematic Reviews of Interventions [15] in our evaluation of the methodology and hence bias risk of the trials. We assessed the bias risk in respect to generation of the allocation sequence; allocation concealment; blinding; intention-to-treat analysis; dropouts; reporting of outcome measures; economic bias; and academic bias. These components enable classification of the included trials into trials with ‘low risk of bias’ or with ‘high risk of bias’. The trials were overall classified as ‘high risk of bias’ if one or more of the above components was ‘uncertain’ or ‘high risk of bias’ [15], [27]–[29]. This classification is important because trials with ‘high risk of bias’ may overestimate positive intervention effects and underestimate negative intervention effects [15], [27], [28], [30], and we wanted to relate the validity of our results to the risk of bias in the included trials.

### Primary outcome measures

#### Depressive symptoms

Our primary outcome was the mean value on the Hamilton Rating Scale for Depression (HDRS) [31], Beck Depression Inventory (BDI) [32], or Montgomery-Asberg Depression Rating Scale (MADRS) [33] at follow-up. We included data based on the total number of randomized patients (intention-to-treat analysis) if these data were reported. We planned to estimate the therapeutic follow-up responses at two time points:

- At cessation of treatment: The trials original primary choice of completion date was used. This was the most important outcome measure time point in this review.
- At maximum follow-up.

#### Adverse events

We classified adverse events as serious or non-serious. Serious adverse events were defined as medical events that are life threatening; result in death; disability or significant loss of function; that cause hospital admission or prolonged hospitalization; a hereditary anomaly; or fetal injury [34]. All other adverse events (that is, events that have not necessarily had a causal relationship with the treatment, but that resulted in a change in or cessation of the treatment) were considered as non-serious events.

#### Quality of life

We included any measure of quality of life, noting each assessment measure.

### Secondary outcome measures

#### Participants without remission

The proportion of participants not having achieved remission was our first secondary outcome. We included data based on the total number of randomized participants (intention-to-treat analysis) - if possible. If the results were not based on the total number of participants, we performed an intention-to-treat analysis assuming that the participants not included in the results did not achieve remission [15]. We pragmatically defined remission as HDRS of less than 8, BDI less than 10, or MADRS less than 10 [31]–[33].

#### Participants with suicidal inclination

Number of suicides, suicide attempts, or suicide inclination was other secondary outcomes.

### Statistical methods

This meta-analysis was undertaken according to the recommendations stated in The Cochrane Handbook for Systematic Reviews of Interventions [15]. In analyzing continuous outcomes with both fixed-effect and with random-effects models, we used the mean difference (MD) with a 95% confidence interval. We used RevMan version 5.0 [35]. We did not use ‘standardized mean difference’ so each outcome measure was analyzed separately. We did not adjust the outcome variables at follow-up according to the baseline values [15].

We used the risk odds ratio (OR) with a 95% confidence interval to estimate intervention effects on dichotomous outcomes with both fixed-effect and with random-effects models [35].

We performed ‘test of interaction’ [36] for all subgroup analyses [18].

For primary outcome measures, we also conducted trial sequential analyses. In order to calculate the required information size and the cumulative Z-curve’s eventual breach of relevant trial sequential monitoring boundaries [16], [17], the required information size of the trial sequential analysis was based on a type I error of 5%, a beta of 20% (power of 80%), the variance of all the trials (as no trial had low risk of bias), and a minimal relevant difference on two points on the HDRS or four points on the BDI.

## Results

### Search results

Our primary literature search identified 4536 publications. According to our protocol [18] we excluded 4137 publications on the basis of the title or abstract, and further 339 citable units were excluded on the basis of the full publication. These exclusions were done either because the publications did not relate to cognitive therapy and major depressive disorder, or because they were not randomized trials comparing cognitive therapy versus ‘treatment as usual’. Further 42 publications [37]–[78] were excluded because the trial participants or the interventions did not meet our inclusion criteria.

### Included trials

We included 18 publications [79]–[96] on eight trials [14], [79]–[82], [84], [85], [97] randomizing a total of 719 participants (**see Figure S1**).

Only five of the trials used an intervention that we classified as ‘adequately defined’ (see above) [14], [79], [81], [82], [97]. We classified the therapists’ level of experience and/or education in three trials as ‘high’ [14], [79], [81], in two trials as ‘intermediate’ [84], [85], and in the last three as ‘unclear’ [80], [82], [97].

Two trials used cognitive group therapy as experimental intervention [80], [82], and one trial used both group and individual therapy [97]. The remaining five trials used only individual therapy [14], [79], [81], [84], [85].

The duration and the extent of the cognitive therapy also varied in the different trials from eight weekly group sessions [80] to 20 weekly individual sessions [14]. The specific content of the ‘treatment as usual’ interventions were generally not standardized or not reported, and the duration and extent of the ‘treatment as usual’ interventions varied greatly between the different trials. Four of the trials allowed antidepressant medication as a part of the ‘treatment as usual’ intervention, but it was not reported to what extent the participants in the four trials received antidepressants. We have described both the experimental and the control interventions from the included trials in **Table 1**.

**Table 1:** Characteristics of the included trials.

Trial	Particiants (randomized)	Interventions	Outcomes and notes
Elkin et al., 1989	124 outpatients	Cognitive therapy (individual, 16–20 weeks) versus pill-placebo and clinical management clinical management: (support, encouragement and advice if necessary)	HDRS, BDI, remission (HDRS<7 & BDI<10)
Scott et al., 1992	60 outpatients	Cognitive therapy (individual, 16 weeks) versus general practitioner care (general practitioner were asked to manage participants as they normally would, including referral to other agencies)	HDRS, remission (HDRS<7)
Embling et al., 2002	38 outpatients	Cognitive therapy (group, 8 weeks) antidepressants versus clinical management+ antidepressants antidepressant: not reported clinical management: weekly 10–20 min sessions	BDI
Miranda et al., 2003	179 outpatients	Cognitive therapy (group or individual, 8–16 weeks) versus community care. Community care: education about depression and mental health treatments available	HDRS, remission (HDRS<8+50%) change from baseline). Participants were low-income young minority women
Verduyn et al., 2003	75 outpatients	Cognitive therapy (group, 16 weeks) versus ‘routine services accessible to participants’	HDRS, BDI
DeRubeis et al., 2005	120 outpatients	Cognitive therapy (individual, 16 weeks) versus placebo pill+clinical management. Clinical management: 10 sessions during 16 weeks	HDRS, remission (HDRS<8) means and SD not included
Dimidjian et al., 2006	98 outpatients	Cognitive therapy (individual, 16 weeks) versus 8 weeks of clinical management+pill placebo. Clinical management: 6 sessions of 30 minutes	HDRS, BDI
Wiles et al., 2008	25 outpatients	Cognitive therapy (individual, 12–20 weekly sessions) versus usual care. Usual care: no restrictions on the treatment that patients could receive	BDI, quality of life means and SD not included. All of the participants had not responded to antidepressants prior to randomization

One of the included trials used antidepressants as add-on therapy in both the experimental group (cognitive therapy) and the control group (clinical management) [80].

Miranda et al. examined the effect of cognitive therapy versus community care [97]. The results showed that cognitive therapy significantly reduced depressive symptoms at cessation of treatment compared with community care. However, the authors did not report means and SD and did not report data on remission rates at cessation of treatment. We have written to the authors requesting the necessary data, but have received no answer. Therefore, we have not been able to include the results from this trial in the following meta-analyses. However, the authors did report rates of remission at six and 12 months follow-up. The results at six months showed no significant difference regarding remission. The results at 12 months showed that cognitive therapy significantly increased the probability of remission compared with community care (P = 0.01).

DeRubeis et al. examined the effect of cognitive therapy versus clinical management plus a placebo pill [81]. The results did not differ significantly regarding HDRS score midway through the intervention period. The authors did not report means and SD, and did not include assessment at cessation of treatment for these outcome measures. We have written to the authors requesting the necessary data, but have received no answer. Therefore, we have not been able to include the results from this trial in the following analysis.

Wiles et al. included participants from primary care who had not responded to at least six weeks of antidepressant medication [85]. The participants were randomized to cognitive therapy versus ‘usual care’. Those who received cognitive therapy had a mean on BDI 9 points lower than those in the ‘usual care’ group, suggesting a beneficial effect of cognitive therapy compared with ‘usual care’. The authors did not report means and SD in the publication. Through e-mail correspondence the authors kindly reported that they were unable to provide the necessary data, so we have not been able to include the results from this trial in the following analysis.

**Table 1** summarizes the characteristics of the eight included trials.

### Bias risk

We assessed all eight trials [14], [79]–[82], [84], [85], [97] as having ‘high risk of bias’ due to unclear or inadequate components as described in **Table 2**.

**Table 2:** Risk of bias.

	Allocation sequence generation?	Allocation concealment?	Intention to treat analysis?	Blinding?	Comparability of drop-outs in intervention groups?	Free of selective outcome measure reporting?	Free of economic bias?	Free of academic bias?	Overall bias assessment
Elkin et al., 1989	Unclear	Unclear	No	Unclear	yes	Yes	Yes	Unclear	High risk of bias
Scott et al., 1992	Unclear	No	No	Unclear	Yes	Unclear	Yes	Unclear	High risk of bias
Embling et al., 2002	Unclear	Unclear	Yes	Unclear	Yes	Unclear	Unclear	Unclear	High risk of bias
Miranda et al., 2003	Yes	Yes	unclear	Yes	yes	Unclear	Yes	Unclear	High risk of bias
Verduyn et al., 2003	Unclear	Yes	No	Yes	No	Unclear	Yes	Unclear	High risk of bias
DeRubeis et al., 2005	Unclear	unclear	yes	Unclear	yes	Unclear	Unclear	Unclear	High risk of bias
Dimijian et al., 2006	Yes	Unclear	No	Yes	No	Unclear	No	Unclear	High risk of bias
Wiles et al., 2008	Yes	Yes	Yes	Unclear	No	Unclear	Yes	unclear	High risk of bias

### Primary outcome measures

#### Depressive symptoms at cessation of treatment

Four trials assessed HDRS as a continuous outcome measure at cessation of treatment [14], [79], [82], [84]. Four trials assessed BDI at cessation of treatment [14], [79], [80], [82].

#### HDRS

**Figure 1:**
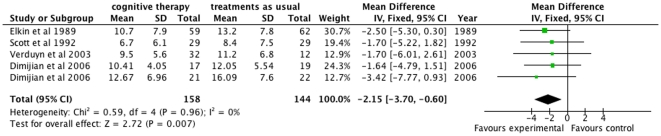
The effect of cognitive therapy versus ‘treatment as usual’ at cessation of treatment on the Hamilton Rating Scale for Depression (HDRS).

Meta-analysis with the fixed-effect model on the HDRS data from the four trials [14], [79], [82], [84] show that cognitive therapy at the end of therapy significantly reduced depressive symptoms compared with ‘treatment as usual’ (**Figure 1**) (mean difference −2.15 HDRS; 95% CI −3.70 to −0.60; P<0.007, I^2^ = 0). The I^2^ statistic describes the percentage of variation across trials that are due to heterogeneity rather than chance. Meta-analysis with the random-effects model gave an identical result.

#### BDI

**Figure 2:**
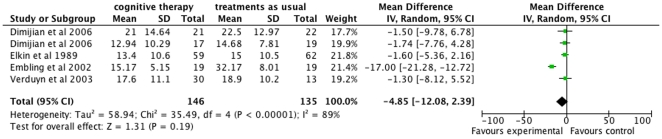
The effect of cognitive therapy versus ‘treatment as usual’ at cessation of treatment on the Beck Depression Inventory (BDI).

Meta-analysis with the fixed-effect model on the data from the four trials [14], [79], [80], [82] using BDI at cessation of treatment was in agreement with the results from HDRS (mean difference −6.03 BDI; 95% CI −8.33 to −3.72; P = 0.00001, I^2^ = 89%). Meta-analysis with the random-effects model showed that cognitive therapy compared with ‘treatment as usual’ did not significantly reduce depressive symptoms on BDI (mean difference −4.85; 95% CI −12.08 to 2.39; P<0.19, I^2^ = 89%) (**Figure 2**). Due to the substantial heterogeneity on the BDI results we performed a sensitivity analysis. We excluded the results from Embling et al. trial and found thereafter no heterogeneity [80]. The possible explanations why the results from Embling et al. differed from the rest of the included trials [14], [79], [82] are discussed below. Meta-analysis with the fixed-effect model on the three remaining trials [14], [79], [82] showed that cognitive therapy compared with ‘treatment as usual’ did not significantly reduce depressive symptoms on the BDI (mean difference −1.57 (95% CL −4.30 to 1.16; P = 0.26, I^2^ = 0). Meta-analysis with the random-effects model gave an identical result.

**Figure 3:**
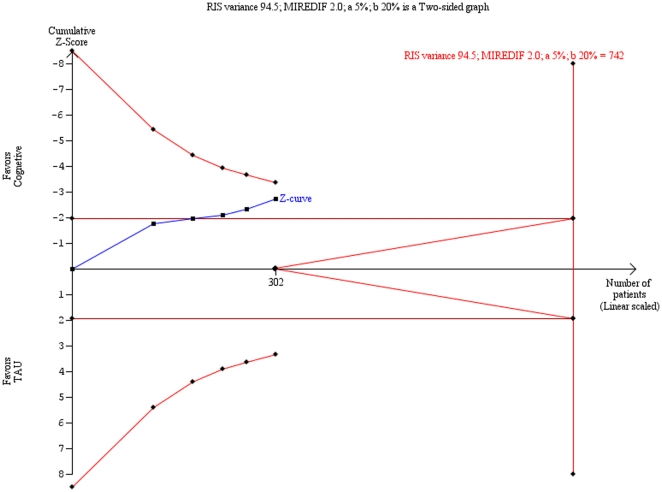
Trial sequential analysis of the cumulative meta-analysis of the effect of cognitive therapy versus ‘treatment as usual’ for major depressive disorder on the Hamilton Rating Scale for Depression (HDRS). The required information size of 742 participants is calculated based on an intervention effect compared with ‘treatment as usual’ of 2 points on the HDRS, a variance of 94.5 on the mean difference, a risk of type I error of 5% and a power of 80%. With these presumptions, the cumulated Z-curve (blue curve) do not cross the trial sequential monitoring boundaries (red inner sloping lines) implying that there is no firm evidence for a beneficial effect of cognitive therapy compared with ‘treatment as usual’.

Trial sequential analysis on the HDRS data and the BDI data showed that ‘insufficient data’ have been obtained to decide if cognitive therapy is superior to ‘treatment as usual’ (**Figures 3** **&** **4**).

**Figure 4:**
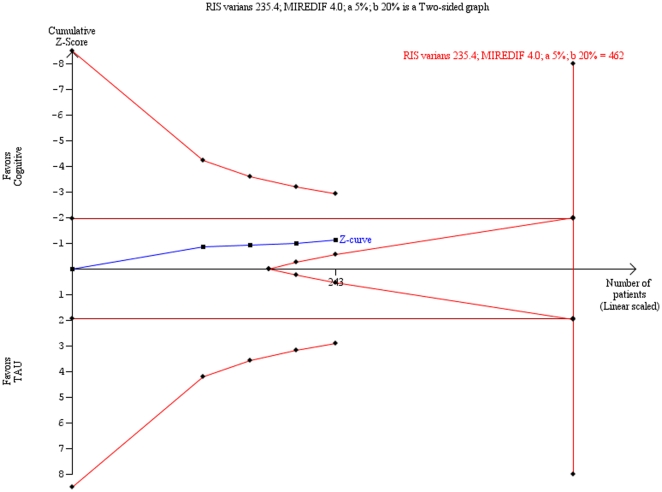
Trial sequential analysis of the cumulative meta-analysis of the effect of cognitive therapy versus ‘treatment as usual’ for major depressive disorder on the Beck Depression Inventory (BDI). The required information size of 462 participants is calculated based on an intervention effect compared with ‘treatment as usual’ of 4 points on the BDI, a variance of 235.4 on the mean difference, a risk of type I error of 5% and a power of 80%. With these presumptions, the cumulated Z-curve (blue curve) do not cross the trial sequential monitoring boundaries (red inner sloping lines) implying that there is no firm evidence for a beneficial effect of cognitive therapy compared with ‘treatment as usual’.

#### Follow-up

Verduyn et al. included maximal follow-up assessment at 12 months after the beginning of treatment on HDRS and BDI [82]. They found no significant difference between the different intervention groups on either of outcome measures.

Miranda et al. reported rates of remission at six and 12 months follow-up [97]. The results are described under ‘Included trials’.

None of the remaining trials included assessment data after the cessation of treatment.

#### Adverse events

DeRubeis et al. reported that two participants dropped out due to adverse events, but the particulars about the events were not reported [81]. Both participants were from the control group receiving placebo. None of the remaining trials reported on adverse events.

#### Quality of life

Wiles et al. assessed quality of life as outcome measure [85]. They found no significant difference between the two intervention groups at cessation of treatment. Means, SD, or choice of outcome measure for quality of life was not reported. None of the remaining trials used any assessment of quality of life.

### Secondary outcome measures

#### Participants without remission

Two trials [14], [84] reported the proportion of participants without remission at cessation of treatment as a dichotomous outcome measure. We had planned to define remission as HDRS less than 8, BDI less than 10, or MADRS less than 10. However, this was not possible, so we adopted the slightly different definitions used by the two trials. Elkin et al. defined remission in two different ways: as HDRS<7 and BDI<10 [14]. Scott et al. defined remission as HDRS<7 [84].

**Figure 5:**
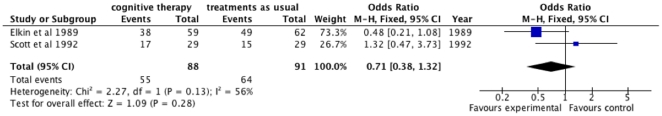
Effect of cognitive therapy versus ‘treatment as usual’ on ‘no remission’ at cessation of treatment.

Meta-analysis on the data from the two trials reporting on HDRS [14], [84] showed that cognitive therapy compared with ‘treatment as usual’ did not significantly decrease the risk of ‘no remission’ (odds ratio of ‘no remission’ in favor of cognitive therapy of 0.71 (95% CI, 0.38 to 1.32; P = 0.28, I^2^ = 56%) (**Figure 5**). The BDI data from Elkin et al. also showed that cognitive therapy compared with ‘treatment as usual’ did not significantly decrease the risk of ‘no remission’ (P = 0.33) [14].

#### Suicide inclination, suicide attempts, or suicides

None of the trials reported on suicide inclination, suicide attempts, or suicides.

### Subgroup analyses

In subgroup analyses stratified according to the type of therapy (group compared to individual therapy) and to the therapists' level of education and experience (‘high’ compared to ‘intermediate’ and ‘unclear’), ‘test of interaction’ [36] on the HDRS data showed no difference in treatment effect between these subgroups (setting P = 0.83; education and experience P = 0.69). Furthermore, we found no heterogeneity in our meta-analysis result on the HDRS data, This indicates that these factors do not seem to influence the effect of cognitive therapy measured on the HDRS.

We had also planned a subgroup-analysis according to risk of bias [18]. However, as all trials were classified as ‘high risk of bias’ it was not possible to conduct this analysis.

## Discussion

The results of our systematic review with meta-analysis (fixed-effect model and random-effects model) indicate that cognitive therapy is likely to significantly reduce depressive symptoms on HDRS compared with ‘treatment as usual’. The result of our meta-analysis after a sensitivity analysis on the BDI data (fixed effect-model and random-effects model) did, however, not show a significant reduction on the BDI. Trial sequential analysis on both on the HDRS data and BDI data showed that insufficient data have been obtained. Finally, cognitive therapy compared with ‘treatment as usual’ did not significantly decrease the risk of ‘no remission’. BDI is a ‘self report’ questionnaire and HDRS is an observer dependant interview. This enables a more objective and blinded assessment of the degree of depressive symptoms with HDRS, but only three trials were assessed as having adequate blinding. We believe the neutral effects on BDI combined with the small effects on HDRS suggest that cognitive therapy may not have dramatic effects.

Trial sequential analysis is a statistical analysis that is adjusting the type I error level to decrease the risk of random errors due to sparse data and multiple testing on accumulating data. Therefore, is a more robust analysis than traditional cumulative meta-analysis [16], [98]. Our analysis demonstrates that we lack firm evidence on the intervention effect of cognitive therapy versus ‘treatment as usual’ for major depressive disorder. The trial sequential analysis result also indicates that in order to detect or reject an intervention effect with a minimal relevant difference of two points on HDRS, an information size of 742 participants may be needed.

The heterogeneity on the results on the BDI data is generated by the results from one trial [80]. The results from the Embling et al. trial showed that cognitive therapy compared with the control intervention, decreased the BDI score with a much greater effect-size than the rest of the trials. Embling et al. was the only of the included trials using antidepressants as add-on therapy as part of both the experimental and control interventions [80]. Although head-to-head comparisons are needed in order to thoroughly examine differences between intervention groups, this finding suggests that adding antidepressants to cognitive therapy might have a greater effect compared to cognitive therapy alone. The Embling et al. trial did only report results on the BDI which is a self report questionnaire hindering a blinded assessment of the depressive symptoms. Furthermore, the trial had only two out of the eight bias risk components classified as ‘low risk of bias’ increasing the risk of biased results. These considerations may support the validity of our post-hoc sensitivity analysis excluding the results from this trial in our meta-analysis.

### Strengths

The present review has a number of strengths. Our protocol [18] was published before we began the systematic literature searches in all relevant databases, data extraction, and data analyses. Data was extracted by two independent authors minimizing the risk of inaccurate data-extraction, and we assessed the risk of bias in all trials according to The Cochrane Handbook for Systematic Reviews of Interventions [15]. We meta-analyzed data both with fixed-effect and random-effects models. Furthermore, we performed trial sequential analysis to control for random errors [16], [98].

### Limitations

Our systematic review has a number of limitations. Only one of the included trials was assessed as being free of ‘selective outcome measure reporting bias’ [15]. There is therefore a risk of within-study selective outcome reporting in seven of the eight included trials. All eight trials had an overall assessment as ‘high risk of bias’ - so our results may be questionable. Moreover, for the positive findings trial sequential analysis showed that we could not exclude the risk of random errors [16], [98]. Due to the limited number of included trials we did not perform a funnel plot or other analysis to explore the risk of publication bias [99]. Other meta-analyses have shown that publication bias significantly has influenced the results from former publications [9]. It is a further limitation that we are not able to assess the risk of publication bias.

Cognitive therapy is generally considered to be one of most evidence-based forms of psychotherapy and we expected to find more randomized trials. However, our literature search did only identify eight trials with a limited number of participants. Only four of the eight trials reported mean and SD for HDRS, and only four of the eight trials reported means and SD for BDI. Our results show that cognitive therapy compared with ‘treatment as usual’ did not significantly decrease the risk of ‘no remission’, but only two out of the eight included trials reported relevant data on remission at end of treatment, while one reported remission rates at six and 12 months follow-up. We might find different results if we had more relevant randomized trials or if we made our inclusion criteria broader (e.g., including trials comparing cognitive therapy with antidepressants).

Only two of the trials included assessments after the cessation of treatment. Therefore it is not clear whether cognitive therapy has an effect on depressive symptoms in the longer term.

Only one of the trials reported measures of quality of life. Outcome measures of quality of life are generally not standardized and thoroughly validated [100]. The use of standardized outcome measures for quality of life in research has been limited by difficulties in administering and scoring quality of life [100], but quality of life can be used as a valid outcome measure [29], [100]. The effect of cognitive therapy on quality of life compared with ‘treatment as usual’ is therefore unclear.

Only one of the included trials reported on some adverse events and none of the trials included records of suicide inclination, suicide attempts, or suicides. Typically adverse events are not reported as thoroughly as beneficial outcome measures [101]. Some psychological interventions might have harmful effects. E.g., psychological debriefing for preventing post-traumatic stress disorder in some clinical trials has showed to have a harmful effect [102]. Possible harmful effects of this kind of therapy are therefore not thoroughly examined.

A number of subgroups of depressed patients (e.g., inpatients) were not assessed in the eight trials we identified and included. These subgroups may react differently to psychotherapy and our results cannot be generalized to other than the patient groups included in the eight trials. Moreover, the extent and form of the ‘treatment as usual’ intervention varied greatly and the specific content of the ‘treatment as usual’ interventions were generally not standardized or reported (**Table 1**). E.g., four of the trials allowed antidepressants as a part of the ‘treatment-as-usual’ intervention but the extent of the antidepressants medication were not reported or controlled for. Due to the unclear content of the control interventions it is possible that the participants in the control groups actually received some kind of psychotherapeutic intervention - possibly including cognitive therapeutic interventions. Furthermore, the duration and extent of the cognitive therapy interventions did also vary in the different trials (**Table 1**). Although head-to-head comparisons are needed in order to thoroughly examine a difference in effect between two interventions, we found no heterogeneity on our results on either HDRS or BDI (after sensitivity analysis) indicating that there might be no difference in effect between the different interventions. Moreover, only five of the included trials presented a treatment manual and documented adherence to the treatment manual for the cognitive experimental intervention. The possible difference between cognitive therapy and ‘treatment as usual’ could be due to this manualization rather than to the specific cognitive technics. These aspects are further limitations and make our results less generally applicable.

As mentioned, only five of the included trials used an intervention that we classified as ‘adequately defined’, i.e., using and documenting the use of a therapeutic manual. And although we did not find any heterogeneity on the HDRS data it is imperative in clinical trials that the interventions are adequately defined and described [103]. Factors like personal style, communication skills, and personality of the therapist evidently will influence the way psychotherapy is delivered [104]. It is difficult to describe and control for these subjective factors, and this makes it even more important to relate the therapy to a treatment manual. Otherwise it is unclear what kind of intervention the participants were receiving, and it is difficult to apply any result in clinical practice.

### Implications

Our meta-analysis show that the possible benefit from this relatively extensive treatment compared with ‘treatment as usual’ was only a few points on the HDRS. From a clinical point of view it could be argued that this possible benefit is not clinically relevant - especially if you relate this mean difference to the extent and length of the intervention. Furthermore, the NICE guidelines [105] recommend that a mean difference on 3 on the HDRS are needed in order for a intervention to be considered significantly clinically effective [105]. We found a mean difference on 2.15 on the HDRS. Other meta-analyses have used this definition to judge if an intervention should be considered clinically effective [9].

In our protocol [18] we chose HDRS, BDI, and MADRS as our primary outcome measures because we expected that most trials would only use these assessment measures, and HDRS has in many years been the gold standard to quantify depressive symptoms in clinical trial [106]. Severity of depression as measured by the total HDRS score has failed to predict suicide attempts [107], and some publications have questioned the usefulness of the HDRS and concluded that the scale is psychometrically and conceptually flawed [106]. MADRS and BDI probably correspond to HDRS [108], [109]. We do not know if these scales are able to assess any potential beneficial effects of cognitive therapy. From the patient's point of view, a score on HDRS, BDI, or MADRS is not necessarily a measure of the degree of suffering, and other assessment methods could demonstrate a more or less substantial effect of any given intervention for depression. The HDRS during 40 years has been the gold standard to quantify depressive symptoms in clinical trials [106]. There is a need for trials assessing and reporting more clinically relevant outcome measures. We believe such assessment methods should be reporting on adverse events and suicidal tendencies, or assessment methods that correspond to clinically relevant outcomes seen from the patient's point of view.

Future research should focus on comparing the effect of cognitive therapy versus ‘treatment as usual’ for major depressive disorder. First and foremost such trials should be conducted with longer follow-up, low risk of bias (systematic errors) and low risk of random errors (play of chance) [110]. Such trials should also report on adverse events, suicide inclination, suicide attempts, and numbers of suicides and the specific content of the ‘treatment as usual’-interventions should be reported. There seems to be a need for a new gold standard assessment method other than HRDS to assess depressive symptoms.

### Conclusions

Cognitive therapy might not be an effective intervention for major depressive disorder compared with ‘treatment as usual’. The possible treatment effect measured on the HDRS is relatively small. More randomized trials with low risk of bias, low risk of random errors, and longer follow-up are needed. Future trials should assess the effect of cognitive therapy on adverse events, suicidal tendencies, quality of life, and other clinically relevant outcomes.

### Ethical approval

Not required.

## Supporting Information

Figure S1 **PRISMA flowchart.**
(TIFF)
