# Supplementary figures and images for: The Effects of Cognitive Therapy Versus ‘Treatment as Usual’ in Patients with Major Depressive Disorder
Source: PLoS One. 2011 Aug 4;6(8):e22890. doi: 10.1371/journal.pone.0022890 (PMC3150380; doi:10.1371/journal.pone.0022890)

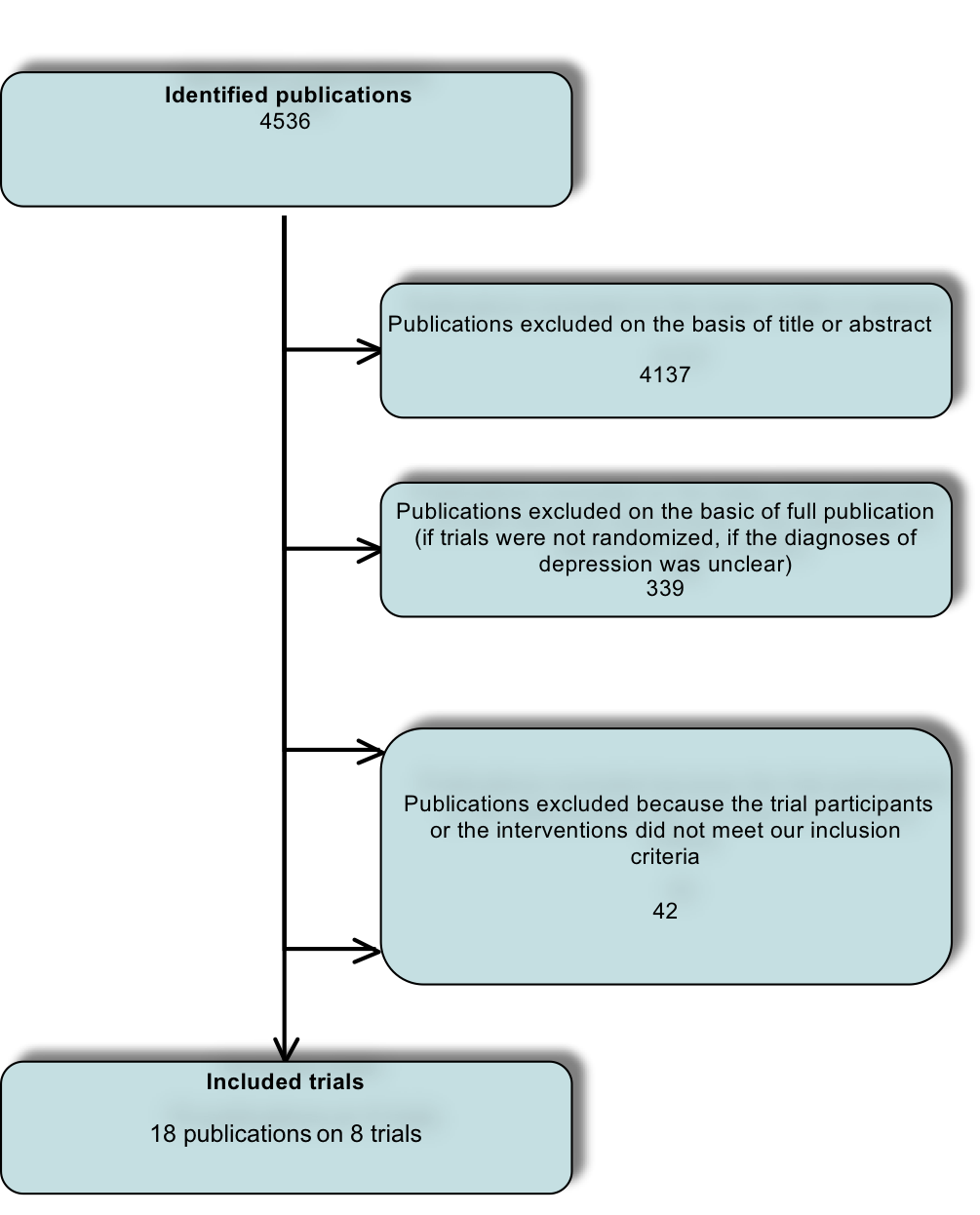

Supplement: Figure S1 — PRISMA flowchart. (TIFF) [file pone.0022890.s001.tiff]
